# Supplementary material for: Robustness of anthropogenically forced decadal precipitation changes projected for the 21st century
Source: Nat Commun. 2018 Mar 20;9:1150. doi: 10.1038/s41467-018-03611-3 (PMC5861119; doi:10.1038/s41467-018-03611-3)
Supplement: Supplementary file 1 — Supplementary Information(PDF 13720 kb) [file 41467_2018_3611_MOESM1_ESM.pdf]

Robustness of Anthropogenically Forced Decadal Precipitation Changes Projected for the 21st Century

Zhang et al.

Supplementary Information

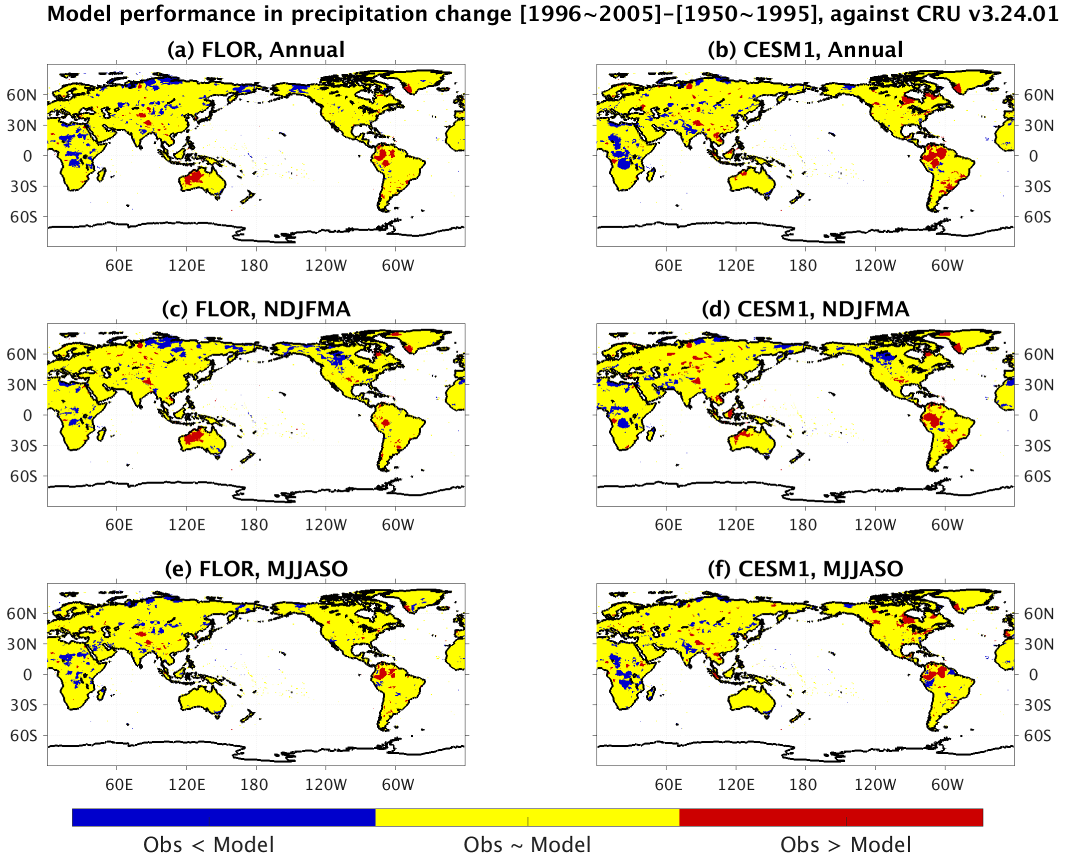

Supplementary Figure 1. Model performance in historical changes of global land precipitation between 1996-2005 and 1950-1995 against the CRU observations. (a, c, e) FLOR and (b, d, f) CESM1 for annual (a, b), NDJFMA (c, d) and MJJASO (e, f) mean precipitation, respectively. Models are considered to be consistent with observations (yellow color) when observed changes are within the range of those simulated by the 35 members of the ALLFORC ensemble. Blue (red) color indicates that observed changes are smaller (larger) than all simulated changes from the 35 ALLFORC members. The inconsistency over few scattered regions (blue and red color) is mostly not systematic across seasons (i.e., does not appear in both seasons) and can arise from a number of reasons: (1) poor model performance, (2) not large enough ensemble size, and (3) observational errors. Overall, both FLOR and CESM1 simulate historical changes in precipitation that are consistent with the CRU observations over most of the global land, which increases our confidence in the utility of the models in investigating the distinguishability of anthropogenic changes in precipitation mean state.

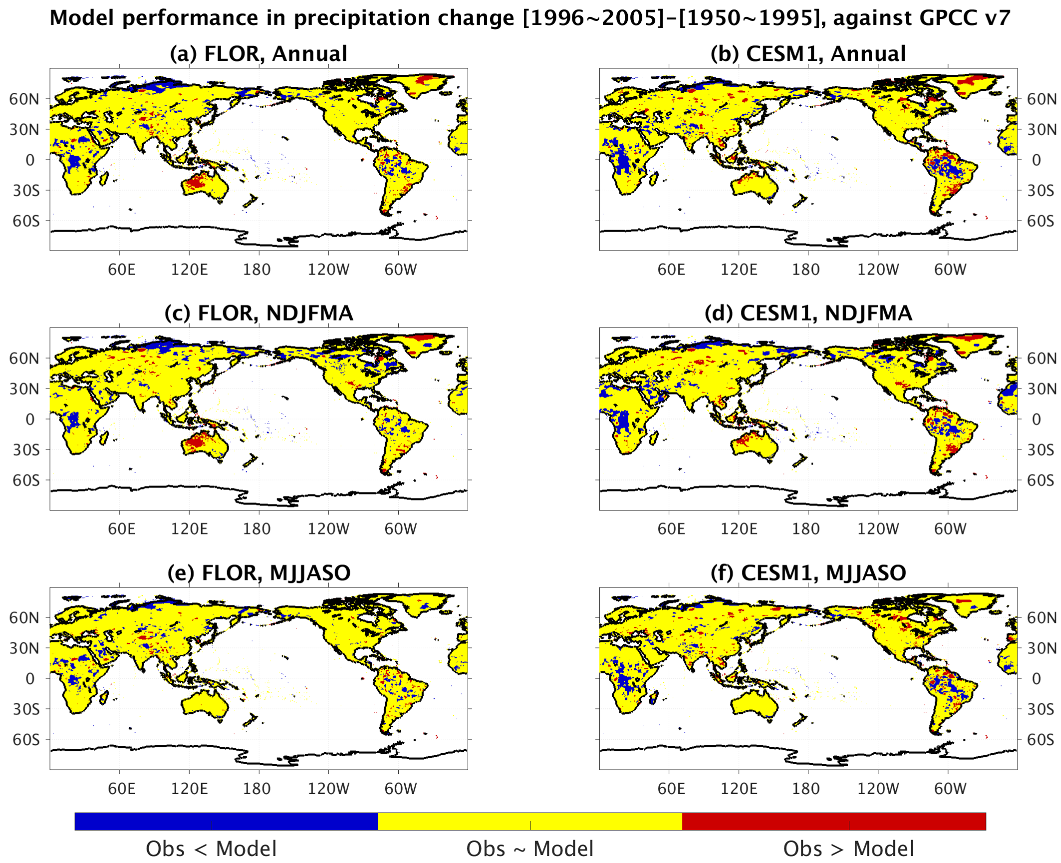

Supplementary Figure 2. The same as Supplementary Fig. 1 but against the GPCC observations. Both FLOR and CESM1 are consistent with the GPCC observations over most of the global land (yellow color), thus increasing our confidence in the utility of the models in investigating the distinguishability of anthropogenic changes in precipitation mean state.

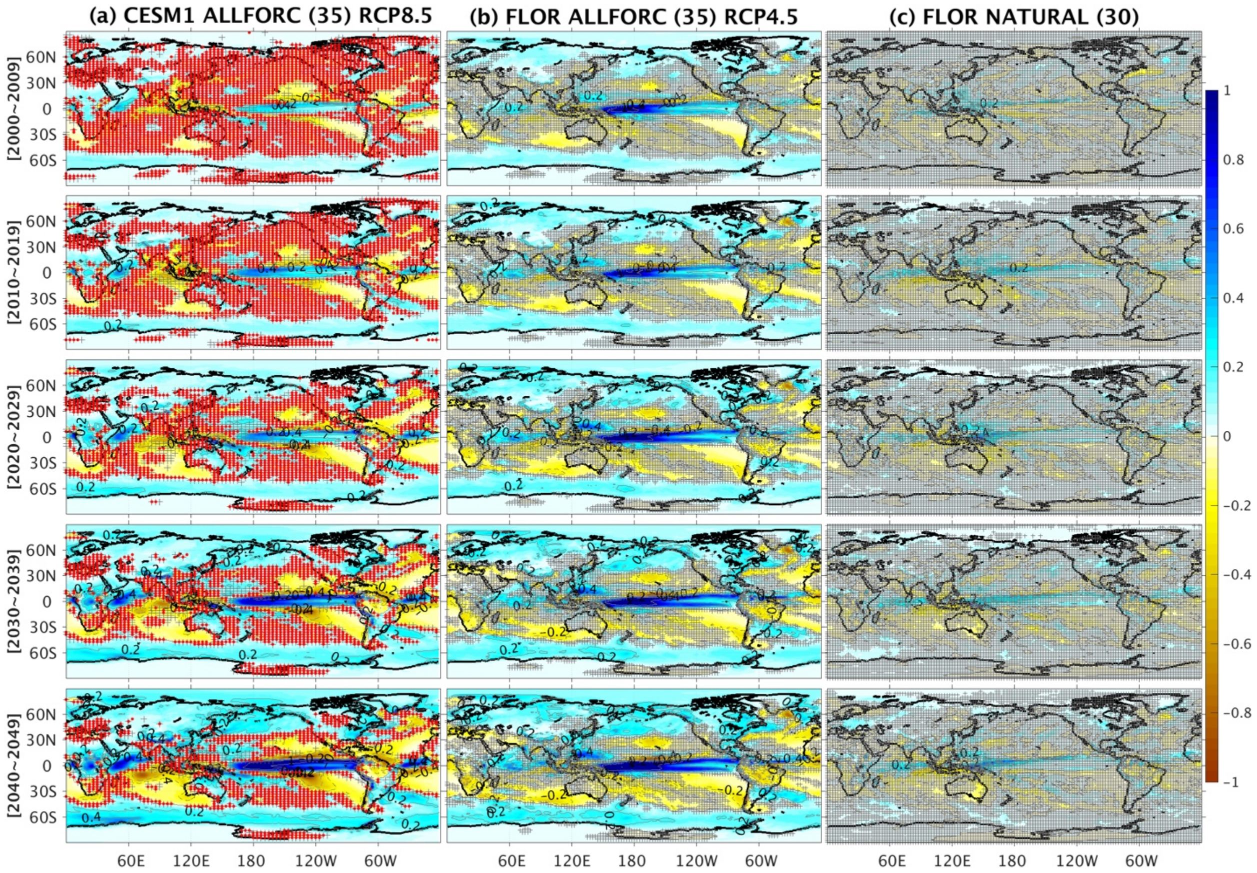

Supplementary Figure 3. Decadal evolution of changes in annual precipitation mean state relative to 1950-1999 during 2000-2050. (a) CESM1 ALLFORC RCP8.5 (35 members), (b) FLOR ALLFORC RCP4.5 (35 members) and (c) FLOR NATURAL (30 members) ensemble average. Time goes down, as denoted to the left of the figure. Contours at intervals of 0.2mm/day are labeled in gray, with dashed denoting negative precipitation changes. Gray crosses in all three columns denote that changes in precipitation mean state are not distinguishable against internal climate variability estimated from fully coupled control simulations (see Methods for details on the distinguishability test); red stippling in (a) denote no distinguishability against internal climate variability estimated from the atmosphere/land-only control simulation of CESM1.

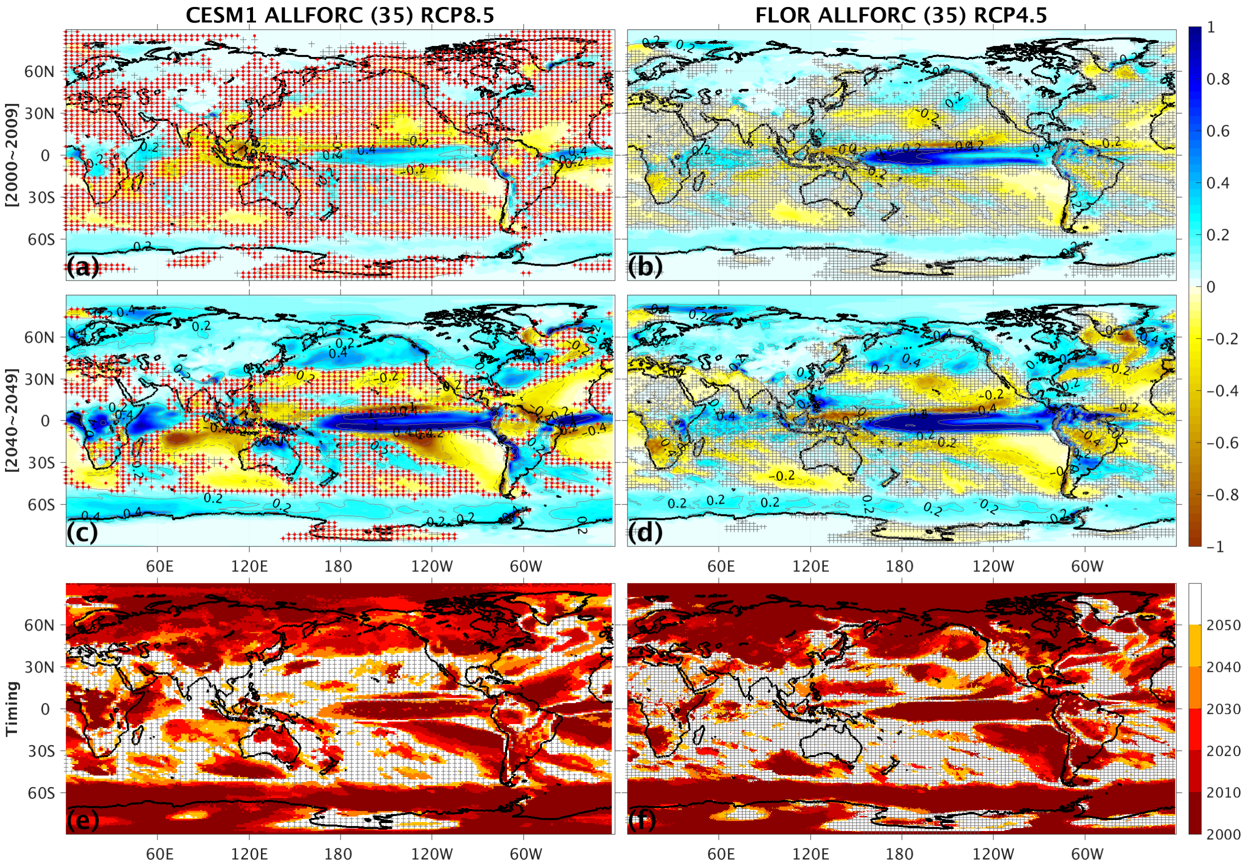

Supplementary Figure 4. Decadal evolution of precipitation mean state and timing of distinguishability in precipitation changes during NDJFMA. (a-d): decadal evolution of changes in wintertime (NDJFMA) precipitation mean state (ensemble average, shading) relative to the 1950-1999 climate during 2000s (a, b) and 2040s (c, d) in CESM1 ALLFORC RCP8.5 (a, c) and FLOR ALLFORC RCP4.5 (b, d). Time goes down, as denoted to the left of the figure (changes during 2010s, 2020s and 2030s are not shown). Contours at intervals of 0.2mm/day are labeled in gray, with dashed denoting negative precipitation changes. Gray crosses in both columns denote that changes in precipitation mean state are not distinguishable against internal climate variability estimated from fully coupled control simulations (see Methods for details on the distinguishability test); red stippling in (a) denotes changes in precipitation mean state are not distinguishable against internal climate variability estimated from the atmosphere/land-only control simulation of CESM1. (e-f): timing of distinguishable changes in precipitation mean state in CESM1 (e) and FLOR (f), defined as the first decade when precipitation changes become distinguishable and remain so thereafter. The gray crosses in (e, f) means no distinguishability by 2050.

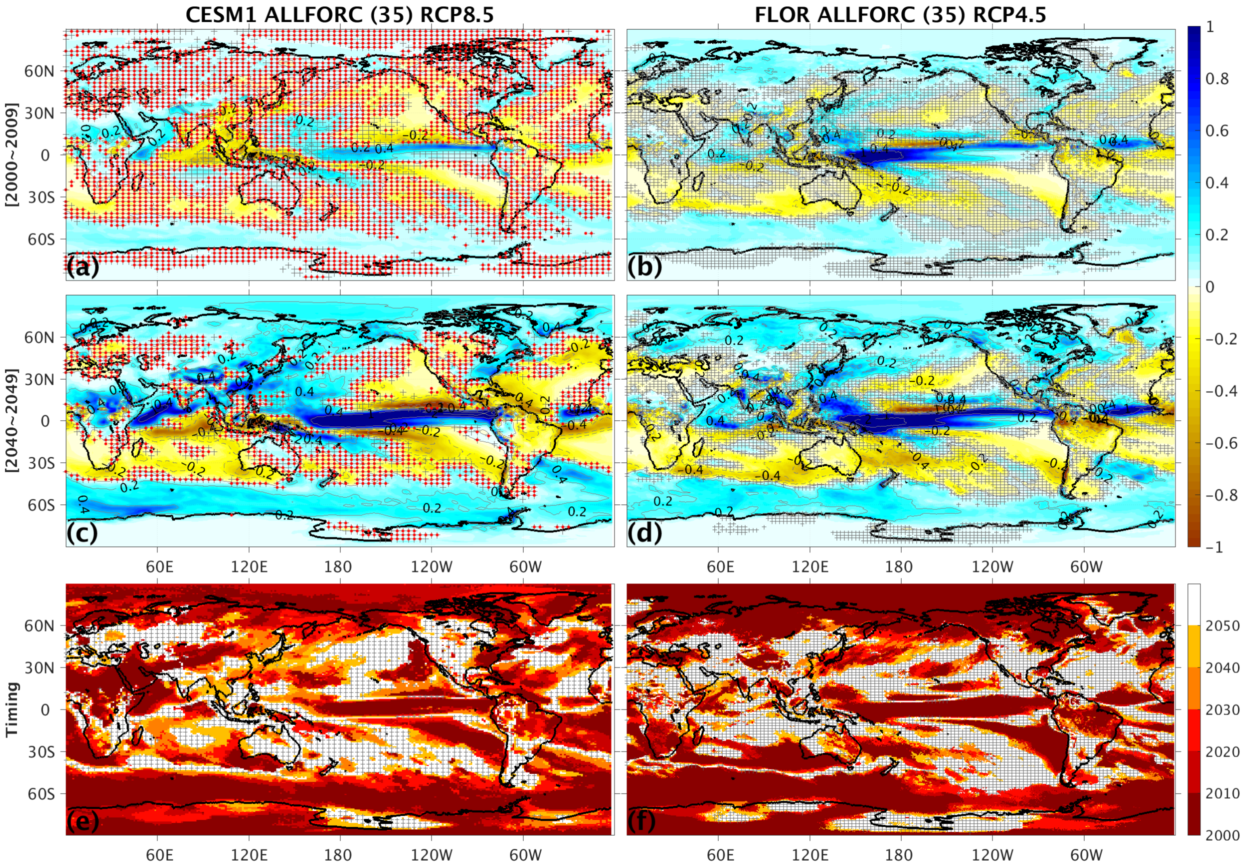

Supplementary Figure 5. Decadal evolution of precipitation mean state and timing of distinguishability in precipitation changes during MJJASO. The same as Supplementary Fig. 4 but for MJJASO.

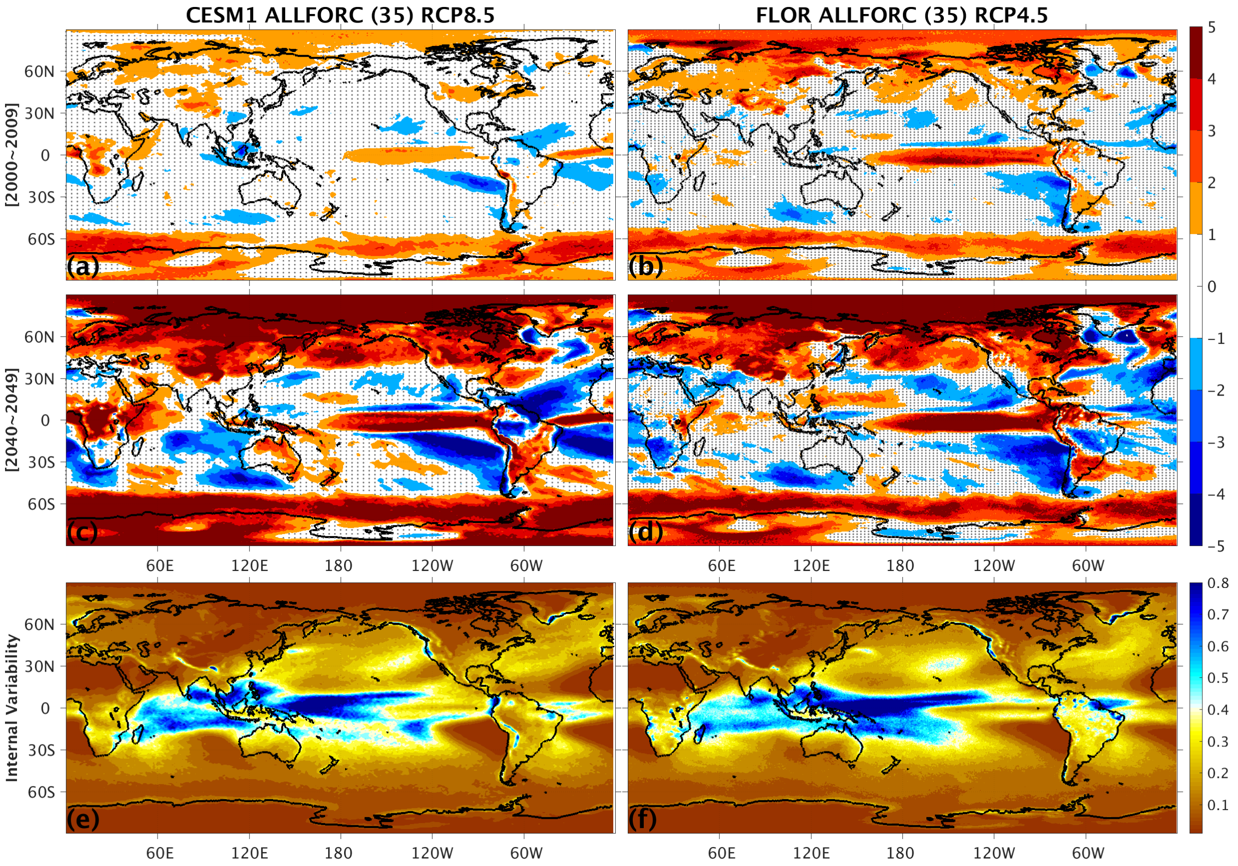

Supplementary Figure 6. Signal to noise ratio and range of the noise for NDJFMA precipitation. (a-d): signal to noise ratio and (e-f): range of the noise (mm/day) for CESM1 (a, c, e) and FLOR (b, d, f), respectively. The signal is the ensemble-mean precipitation change shown in Fig. S4a-d and the noise is the NDJFMA precipitation low-frequency internal variability against which the signal is tested. The range of the noise is estimated as the difference between the maximum and minimum (i.e., the most positive and negative) values of the 5000 samples constructed with the Monte-Carlo approach, where each sample represents the synthetic ensemble-mean precipitation change arising entirely from internal climate variability (see Methods for more details). The signal to noise ratio is computed as the ratio of positive (negative) ensemble-mean precipitation change to the maximum (minimum) value of the 5000 samples. Regions without distinguishability (i.e., signal to noise ratio between 1 and -1) are indicated by white color with gray stippling in (a-d) (showing the decades of 2000-2009 and 2040-2049, respectively).

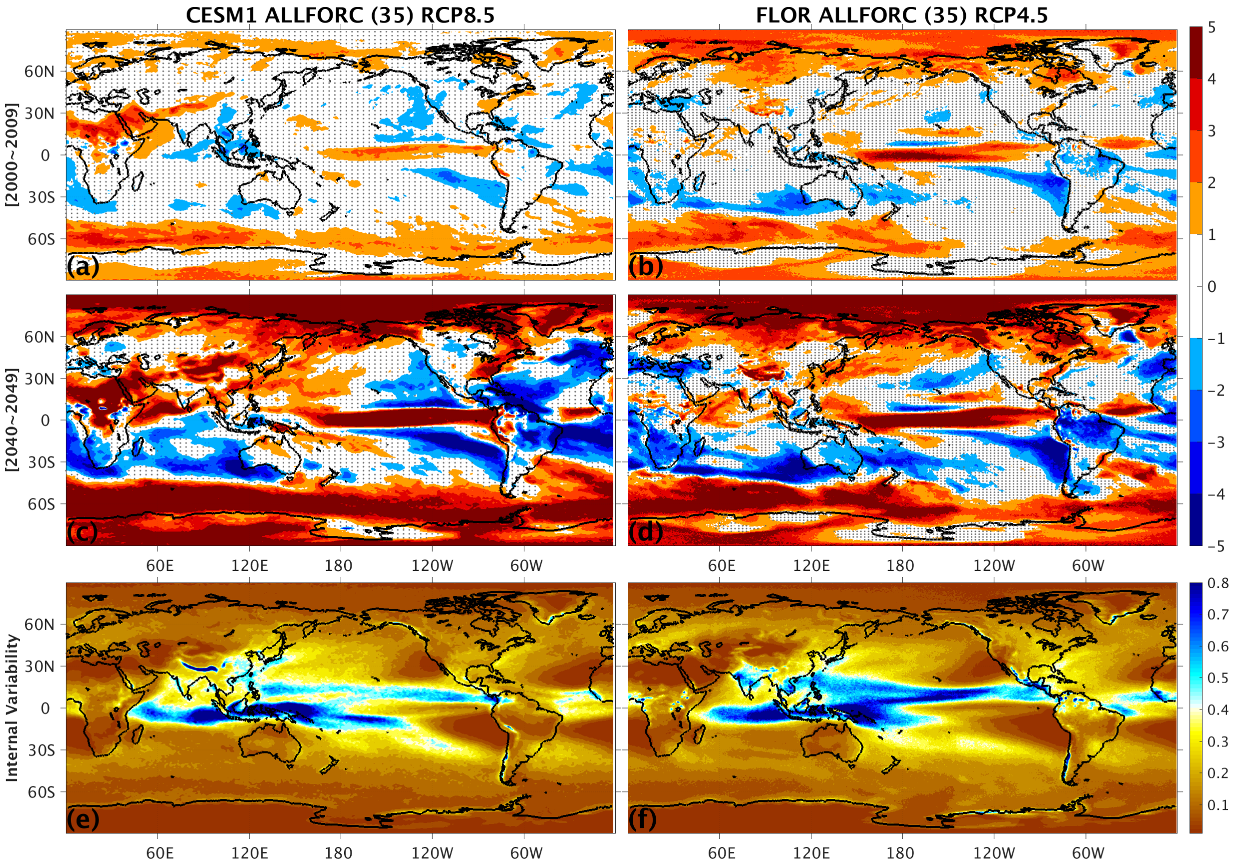

Supplementary Figure 7. Signal to noise ratio and range of the noise for MJJASO precipitation. The same as Supplementary Fig. 6 but for MJJASO.

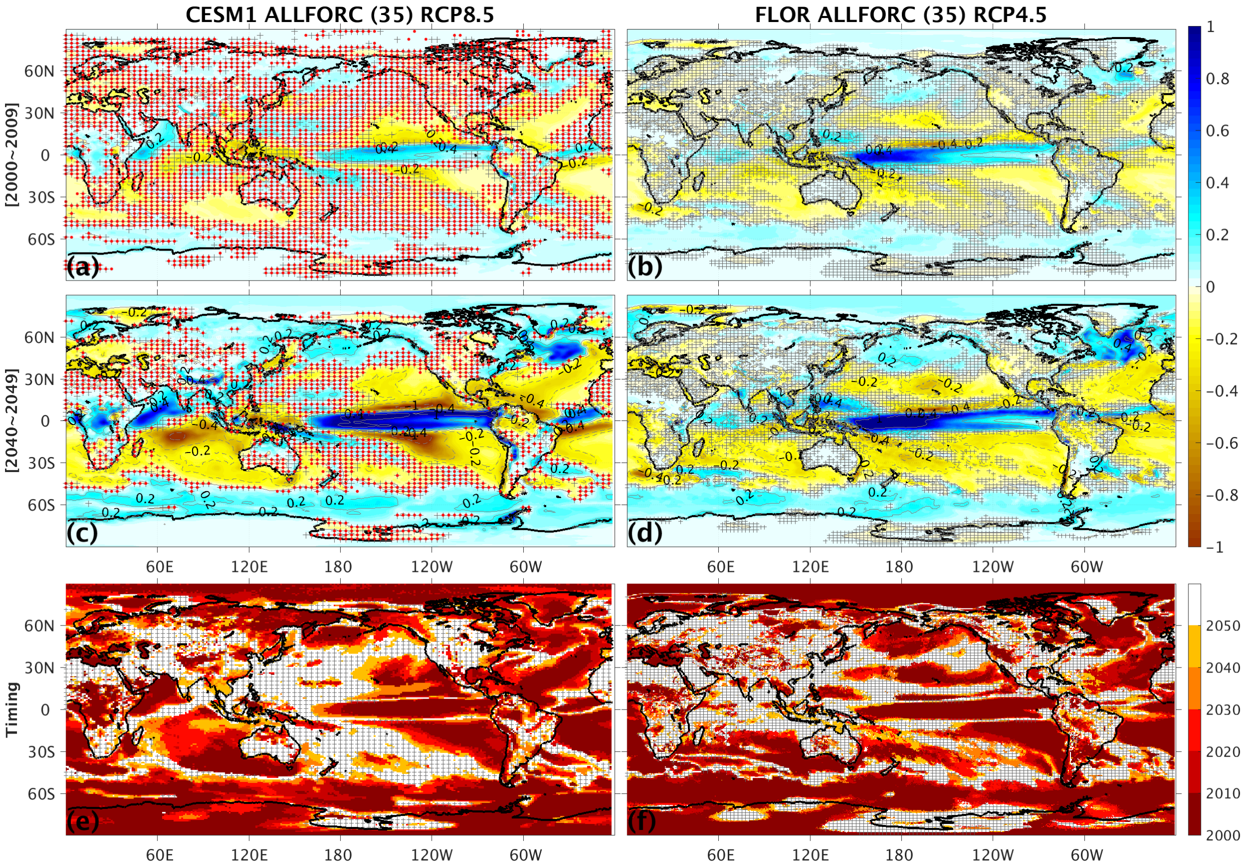

Supplementary Figure 8. Decadal evolution of annual PmE (precipitation minus evaporation) mean state and timing of distinguishability in PmE changes. The same as Fig. 1 in the main text but for PmE.

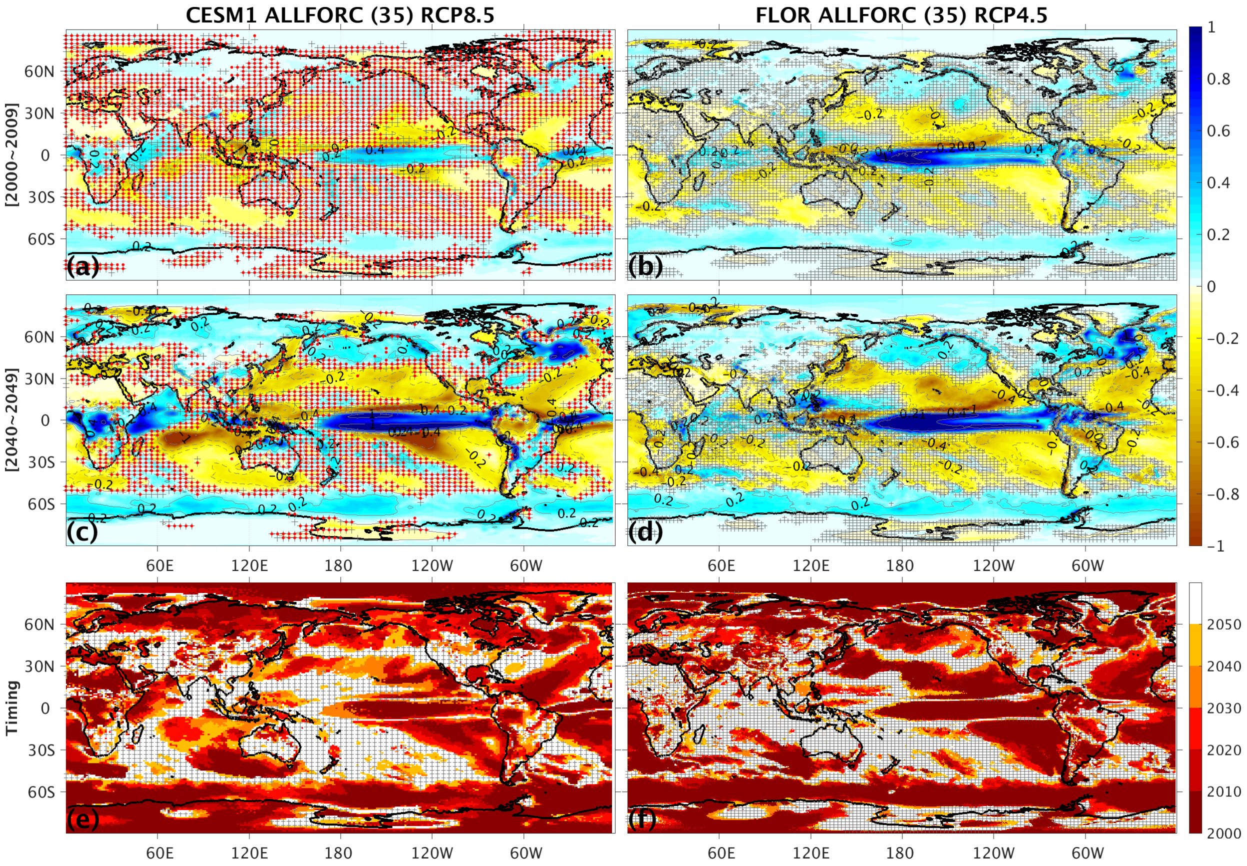

Supplementary Figure 9. Decadal evolution of NDJFMA PmE mean state and timing of distinguishability in PmE changes. The same as Supplementary Fig. 4 but for PmE.

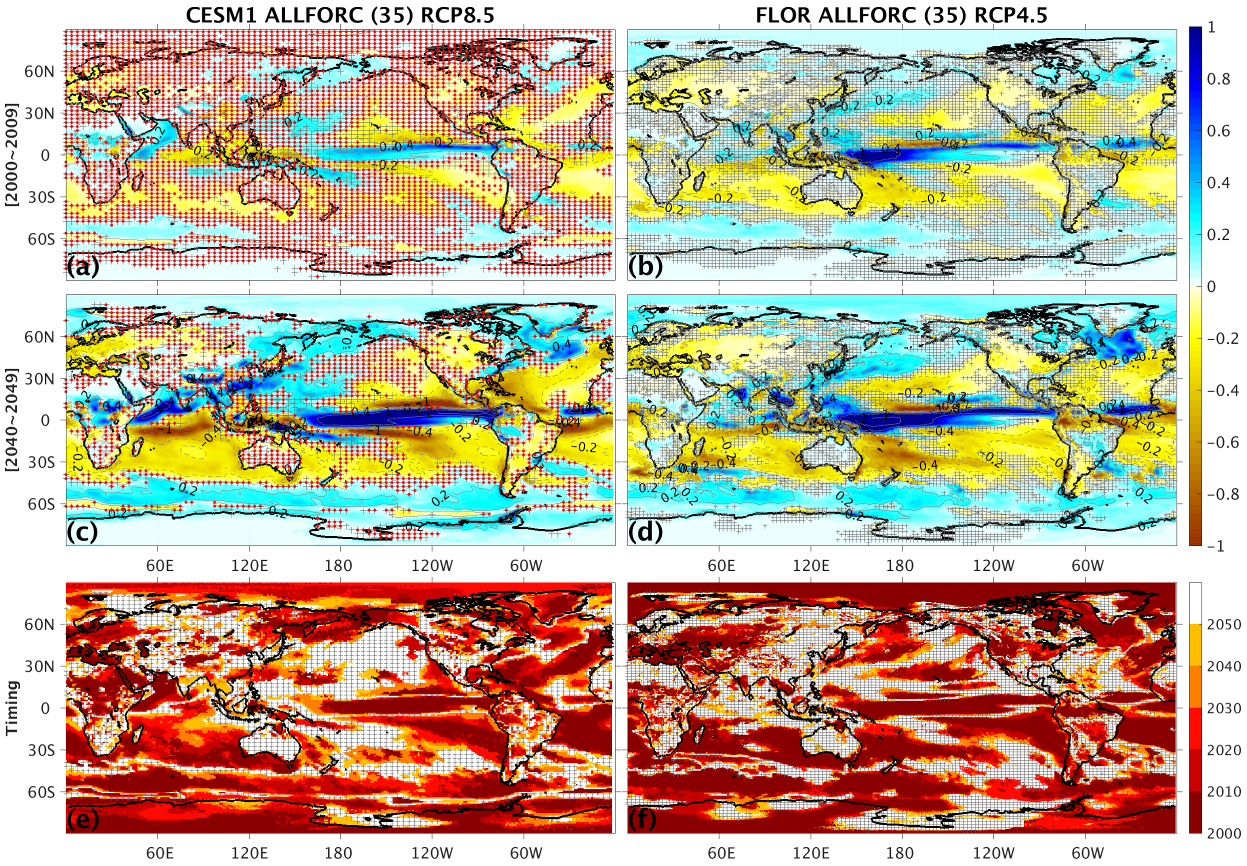

Supplementary Figure 10. Decadal evolution of MJJASO PmE mean state and timing of distinguishability in PmE changes. The same as Supplementary Fig. 5 but for PmE.

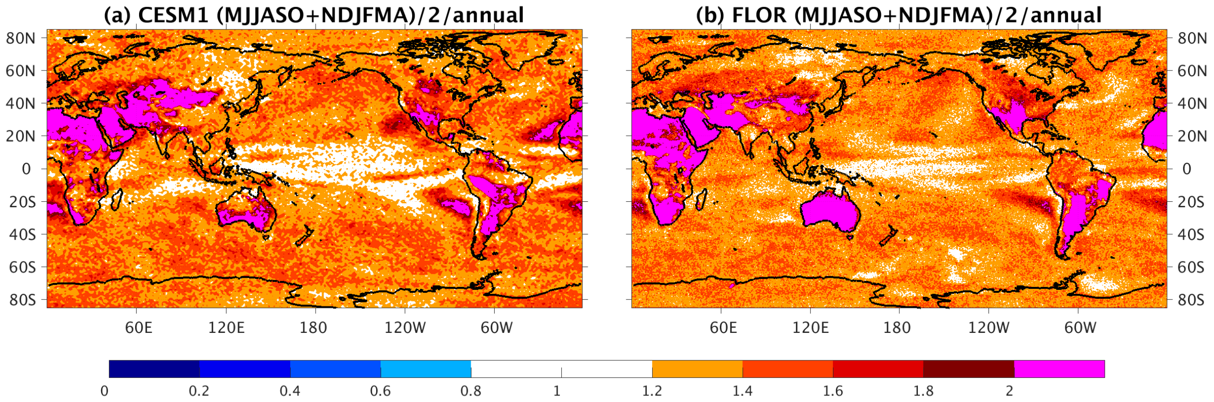

Supplementary Figure 11. Ratio of the average PmE noise between NDJFMA and MJJASO to the annual PmE noise. (a): CESM1 and (b): FLOR. The noise refers to the PmE low-frequency internal variability and is estimated as the difference between the maximum and minimum (i.e., the most positive and negative) values of the 5000 samples constructed with the Monte-Carlo approach from fully coupled control simulations.

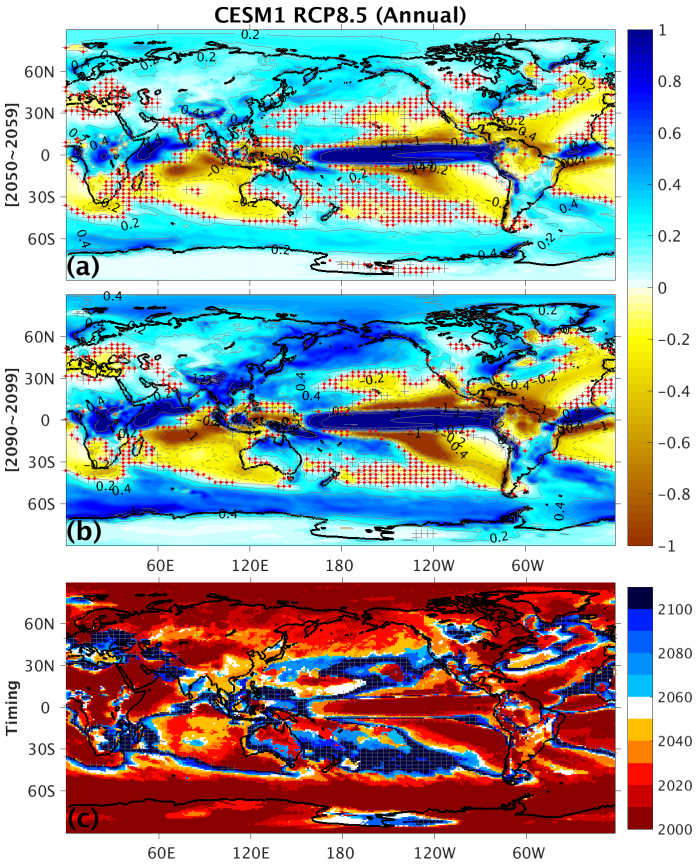

Supplementary Figure 12. Decadal evolution of annual precipitation mean state during 2050-2100 and timing of distinguishability in precipitation changes in CESM1. The same as Fig. 1(a, c, e) in the main text but for 2050-2100. (a) and (b) show the decades of 2050-2059 and 2090-2999, respectively.

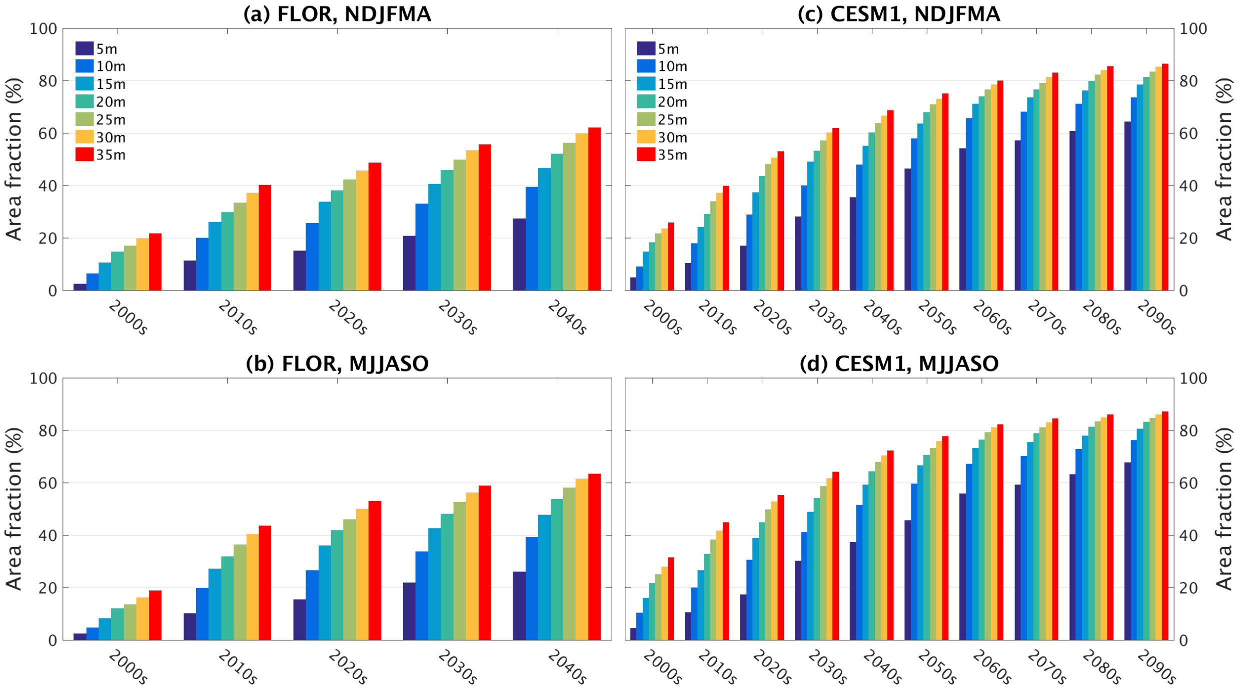

Supplementary Figure 13. Sensitivity of the distinguishability analysis to ensemble size. Fraction of area with distinguishable shifts in precipitation mean state over the whole globe in FLOR (a, b) and CESM1 (c, d) for NDJFMA (a, c) and MJJASO (b, d), respectively. Each color represents a subset or the entire set (red) of the 35-member ALLFORC ensembles (see legends in a, c) used in the distinguishability analysis. The sensitivity to ensemble size gets weaker as the ensemble size grows, especially when it exceeds about 25.

Supplementary Table 1. Fraction (%) of the global area (weighted by latitudes) with distinguishable shifts in annual/NDJFMA/MJJASO PmE mean state, respectively.

	**GFDL FLOR 35-mem ALLFORC RCP4.5**			**NCAR CESM1 35-mem ALLFORC RCP8.5**		
	Land	Ocean	Total	Land	Ocean	Total
2000-2009	22.7/27.1/28.5	40.6/41.2/43.6	35.4/37.1/39.3	26.6/26.0/22.7	34.3/27.1/30.3	31.8/26.7/27.8
2010-2019	32.2/37.8/39.4	52.1/50.5/53.2	46.4/46.8/49.2	38.7/39.0/37.6	47.1/39.8/43.3	44.3/39.5/41.4
2020-2029	39.6/47.1/47.6	63.0/60.2/62.7	56.2/56.4/58.3	48.0/51.9/49.3	58.9/52.9/56.5	55.3/52.5/54.1
2030-2039	46.0/54.2/54.0	66.3/63.9/67.1	60.4/61.1/63.3	57.0/61.1/56.8	65.9/62.4/67.1	63.0/62.0/63.7
2040-2049	49.7/57.5/58.2	71.3/66.9/70.8	65.0/64.2/67.2	62.8/66.8/64.3	72.7/69.0/73.6	69.5/68.3/70.6
2050-2059				68.5/74.1/71.6	76.9/74.2/78.6	74.2/74.2/76.2
2060-2069				72.9/78.8/76.1	81.5/78.2/83.6	78.7/78.4/81.1
2070-2079				77.2/82.2/79.7	84.1/81.3/85.2	81.8/81.6/83.4
2080-2089				77.2/84.0/81.8	86.2/83.0/87.4	83.2/83.3/85.6
2090-2099				79.7/86.1/83.4	86.9/85.0/88.0	84.6/85.4/86.5

The distinguishability of externally forced shifts in PmE mean state (relative to the 1950-1999 mean climate) is estimated against internal climate variability in fully coupled control simulations (see Methods for more information). Results are shown for the two ALLFORC ensembles.
